# Consumer Perception of Beef Quality and How to Control, Improve and Predict It? Focus on Eating Quality

**DOI:** 10.3390/foods11121732

**Published:** 2022-06-13

**Authors:** Jingjing Liu, Marie-Pierre Ellies-Oury, Todor Stoyanchev, Jean-François Hocquette

**Affiliations:** 1 INRAE, Clermont-Ferrand, VetAgro Sup, UMR1213, Recherches sur les Herbivores, 63122 Saint Genès Champanelle, France; jingjing.liu@inrae.fr (J.L.); marie-pierre.ellies@agro-bordeaux.fr (M.-P.E.-O.); 2 Bordeaux Sciences Agro, 1 Cours du Général de Gaulle, CS 40201, 33175 Gradignan, France; 3 Veterinary Faculty, Trakia University, 6000 Stara Zagora, Bulgaria; todor.stoyanchev@uni-sz.bg

**Keywords:** beef quality attributes, beef eating quality, consumer perception, *pre-* and *post-mortem* determinisms, beef grading scheme

## Abstract

Quality refers to the characteristics of products that meet the demands and expectations of the end users. Beef quality is a convergence between product characteristics on one hand and consumers’ experiences and demands on the other. This paper reviews the formation of consumer beef quality perception, the main factors determining beef sensory quality, and how to measure and predict beef eating quality at scientific and industrial levels. Beef quality is of paramount importance to consumers since consumer perception of quality determines the decision to purchase and repeat the purchase. Consumer perception of beef quality undergoes a multi-step process at the time of purchase and consumption in order to achieve an overall value assessment. Beef quality perception is determined by a set of quality attributes, including intrinsic (appearance, safety, technological, sensory and nutritional characteristics, convenience) and extrinsic (price, image, livestock farming systems, commercial strategy, etc.) quality traits. The beef eating qualities that are the most valued by consumers are highly variable and depend mainly on the composition and characteristics of the original muscle and the *post-mortem* processes involved in the conversion of muscle into meat, the mechanisms of which are summarized in this review. Furthermore, in order to guarantee good quality beef for consumers in advance, the prediction of beef quality by combining different traits in scenarios where the animal, carcass, and muscle cuts can be evaluated is also discussed in the current review.

## 1. Introduction

Beef quality is of paramount importance to consumers since consumer perception of quality determines the decision to purchase and repeat purchase, which is of utmost importance for the development and success of the beef market and industry. Beef quality is multifactorial, and consumer perception of beef quality mainly depends on four dimensions: (1) search quality (visual appeal): the morphological property of beef such as the appearance (e.g., color, visible fat), technological, and convenience quality attributes (e.g., cooking yield, shelf life); (2) experience quality (sensory appeal): the eating experience of beef such as beef tenderness, juiciness, and flavor liking; (3) credence quality: the safety, nutritional, and health value of a product and other additional values related to certain attributes such as animal welfare and environmental sustainability; (4) quality of value (cost effectiveness): the cost/price of the product is perceived to correspond as much as possible to the value and image of the product [1,2].

Prior to purchase, only search quality can be reached by consumers, and based on the appearance of the product, consumers might develop expectations according to the available information conveyed by the extrinsic cues of a product at the time of purchase. Extrinsic quality cues primarily have their influence on the search and credence quality of the product from the outside, such as brand, origin, price, and image [1]. The image value is more related to the livestock and industrial production system [1], which would create an overall image of the product presented to consumers.

On the other hand, the experienced quality is the key criteria that is most responsible for the actual demand and satisfaction of consumers and their repeat purchase intention [3]. However, experienced quality can only be determined after purchase and is mainly related to the intrinsic properties of the product. These intrinsic factors cover the physical characteristics of the beef product itself, such as meat color, muscle cut, fat content and marbling distribution, [4] etc.

**Figure 1:**
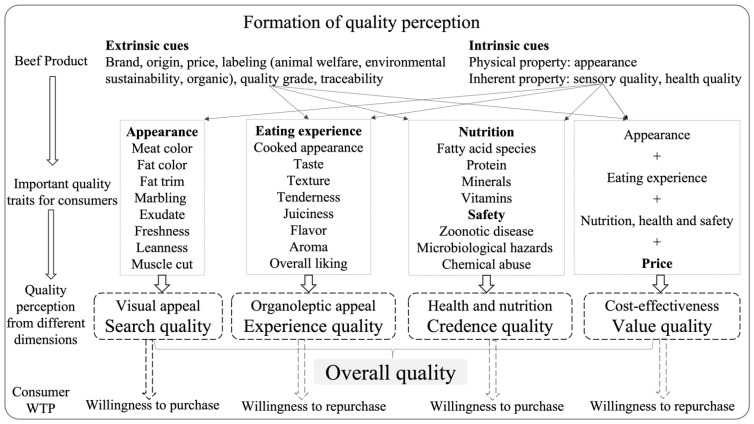
The formation of consumer beef quality perception.

Quality is sought because it contributes to the fulfillment of purchase motives [1]. Consumers expect good quality when they eat beef, but the presumed and/or experienced eating quality may not always match their expectations and the price they paid is usually higher than what the product actually deserved [5]. This is, at least in part, the major reason why beef consumption has declined, especially in Europe [6]. Maintaining consistency between expectations and actual experience is beneficial to a long-term consistent consumption level and to the success of the beef industry. The study of consumer decision-making on intrinsic and extrinsic cues is essential in order to understand how consumer quality perception for beef products is formed (Figure 1). More precisely, consumers’ inability to predict their own quality experience after purchase is sometimes due to the scarcity of extrinsic cues and misinterpretation of intrinsic cues. For instance, a higher marbling level may not be good for the credence quality for normal consumers, but it is actually good for the eating quality of the product [1,2]. In this situation, an appropriate extrinsic cue is needed to inform consumers of the relevant eating quality through the visible marbling. This indicates that the perception of beef quality is affected by both intrinsic and extrinsic quality cues, and the perceived intrinsic cues are affected by the perceived extrinsic cues and vice versa. This is useful for the industry to align the value more accurately with the quality of the product for marketing purposes.

Much scientific and industrial effort has therefore been devoted to closing the gap between expectation and experience through the consistency of extrinsic (e.g., brand, grade, price, etc.) and intrinsic (e.g., marbling level, eating quality, etc.) cues of the product and aligning value as closely as possible with the quality of the product. The Meat Standards Australia (MSA) system is a good example. In this system, beef eating quality is consistently guaranteed by a specified quality grade and a money-back guarantee. Indeed, the quality of beef that consumers receive is that for which they are prepared to pay [2] that ensures consumer satisfaction to large extent. In addition, the price premiums generated predominantly from the MSA-guaranteed quality are being noticed by the Australian beef industry [6]. Overall, understanding quality factors and ultimately improving beef quality is imperative to both consumers and the industry.

The aims of this review article are therefore to first describe consumers’ perception of beef quality, then the main factors affecting beef eating quality, and finally the main methods of measurement and prediction of beef eating quality.

## 2. Consumer Perception of Beef Quality

### 2.1. Extrinsic Quality Traits

When consumers select products in shops and markets, the extrinsic cues conveyed by the products play a dominant role in the formation of quality perception and expectation and in the subsequent purchase decision-making [1]. Within the meat sector, the extrinsic quality traits of beef are predominantly related to production, processing, and marketing, including the commercial quality of beef carcasses, brand, origin, image, quality grade, price and other product information that is of value to consumers, such as animal breed, feeding resources, breeding environment, and ethical, cultural, and environmental dimensions [7].

#### 2.1.1. Commercial Quality, Quality Grade and Price

The variability in bovine carcasses and, consequently, in meat quality is high, inconsistent, and multifactorial in origin [7]. As the unit of trade and grading is based on carcasses, the commercial quality of bovine carcasses is of paramount importance not only for farmers, but also for the intermediate actors of the supply chain and retailers to ensure an optimized meat quality for consumers [8]. In many countries such as the United States, Japan, and South Africa, the commercial quality of a carcass is mainly evaluated based on animal type (sex and age) and carcass weight. Additionally, conformation and fat scores are important commercial quality traits of carcasses in European countries. Other attributes can indicate carcass quality, especially with the incorporation of meat quality predictors, but these attributes are also carcass-based, which means they can be considered as commercial quality attributes of the beef carcass. With the exception of sex and carcass weight, other traits such as rib fat depth, marbling score, ultimate pH, ossification, and hump height (estimate of *Bos indicus* content) are used by MSA to predict beef eating quality. All of these attributes related to carcass quality can be good references for producers to target the value proposition between commercial carcass quality and eating quality [2]. For instance, as the marbling score is a key component of beef quality, there are several mechanisms available to breeders to improve this attribute through genetics, animal type, carcass weight and fat level, high-energy diets, and maturity patterns, to ultimately improve the quality of beef products for consumers [9].

There are several beef grading schemes (i.e., MAS, USDA, JMGA) around the world to score beef eating quality. It has been shown that quality grade is a reliable predictor of expected quality in studies on consumer willingness to purchase. Indeed, Lyford et al. (2010) found that consumers from different countries (Japan, Australia, the United States, and Ireland) would be willing to pay more for beef with a higher quality grade [10]. This implies that a higher grade is linked to a better quality perception and that consumers would indeed be willing to pay a premium for the product that is perceived as valuable to them. On the other hand, price is also used as an indicator of quality since a higher price should logically correspond to a higher quality. Unlike the predominant extrinsic cues such as brand and quality grade, which are associated to a large extent with experience and credence quality, price only influences credence quality expectations of consumers [3]. In fact, price may be more related to the packaging characteristics of the product in certain circumstances, and is also a strong driver of perceived quality [11]. It has also been observed that in France, there is no apparent link between the market price of beef and the perceived tenderness of the meat by consumers. This implies that consumers can either obtain good beef at a low price or be disappointed by expensive beef by pure chance [12]. This emphasizes that an accurate grading scheme for at least beef eating quality is paramount in order to enable value-based purchasing for consumers.

#### 2.1.2. Brand

Specifically, when consumers have prior knowledge of the brand, it is regarded as the most dominant factor in forming expected quality as consumers rely on brand as a trustworthy quality indicator to help them reduce purchase uncertainty due to the high biological variability in beef quality [3]. For example, Certified Angus Beef brand is reputed to be tender, juicier, and flavorful, and indeed receives a higher palatability perception by consumers [13]. Brand is also linked to the image of a product, such as the “Label Rouge” in France, which represents a superior quality in terms of palatability and credence [14]. In addition, the information given by labels could raise consumers’ expectations of extraordinarily high quality. The labelling of a superior fatty acid composition or information that the cattle were raised on natural pasture or certain specialized breeds such as Aberdeen Angus beef, could create a sense of luxury and pleasure among consumers, as they traditionally associate them with good quality [15]. In terms of “luxury”, some beef brands aim to produce highly marbled products (e.g., Japanese Wagyu) with more than 10% or even 20% fat. Despite the fact that these brands are not intended for everyday consumer consumption or the mainstream market, and do not have a strong focus on the health and nutrition issues that consumers value, they still have a market share due to the premium and luxury they are associated with, which can give consumers a sense of social importance when consuming these products [2].

#### 2.1.3. Origin

Origin has often been considered by consumers as an important predictor to product value, while this quality cue does not seem to have an impact on eating quality [16]. In fact, as pointed out by Loureiro and Umberger [17], origin can only become a symbol of superior quality if consumers associate this origin with higher quality and safety. Despite the lack of direct influence of origin on quality, an indirect relationship actually exists through the emotional connection established for consumers that influences perceived intrinsic cues, which in turn influence expected quality [3]. Consumers also believe that local breeds are closer to the terroir [18]. In Europe, quality origin (‘PDO’ for protected designation of origin and ‘PGI’ for protected geographical indication) represents excellence in European agricultural food production and is valued by consumers for the unique combination of human and environmental factors that are based on specific quality characteristics derived from a specific geographical origin [14]. As in 2019, the European Union had a total of 1421 PDO and PGI registered products.

#### 2.1.4. Image

In contrast to monogastric animals, the arguments against beef production do not so much concern food competition with humans but are more related to environmental sustainability in terms of greenhouse gas (GHG) emissions and use of land and water [7]. Another issue in relation to beef production is animal welfare, such as the animal suffering caused by factory farming systems to boost meat productivity. The environmental, ethical, and cultural aspects related to how the product is produced and processed all contribute to the quality perception of consumers. These image value attributes are therefore useful indicators of the quality of a beef product. Since livestock production is considered as a primary source of greenhouse gas emissions, the “low-carbon diet” has become a new trend in the catering sector [19]. A “carbon label” can remind consumers of the GHG impact of the food product. This image has a positive association with consumers’ perception of the quality of the food product [20]. The animal welfare assessment system developed following the European Welfare Quality project could improve the quality of the product image if the assessment can be applied on the products and the information could be provided to consumers. Indeed, animal welfare on-package labels can boost consumer appreciation and purchase intention [21]. Some animal welfare regulations during animal transport and slaughter to reduce animal stress can be a decisive factor for image-related quality attributes. Furthermore, some consumers are more willing to pay for on-farm slaughtered beef products [22].

In fact, image quality is a broad concept since almost all the extrinsic attributes can constitute the holistic image of a product. In other words, consumer perception of food quality at the time of searching/purchasing is based on the overall image of the product, which is indeed a major driver of purchase decision-making [23]. More importantly, consumer perception is also associated with ethical and environmental sustainability, and therefore improving the image value of beef products could be a key strategy for the industry.

### 2.2. Intrinsic Quality Traits

Intrinsic quality traits mainly include three categories: (1) appearance, which is part of the physical characteristics of the product that visually define a given category of beef product such as muscle cut, meat color, fat color, fat trim, marbling, and exudate [24]; (2) sensory quality, which is the perceived overall quality of beef (e.g., eating experience) and preferences for individual sensory responses during beef consumption (e.g., taste, tenderness, juiciness, flavor, aroma, freshness, and leanness) [1]; (3) health quality, which is associated with credence quality, including safety, nutritional composition, and healthiness of the product [25]. In contrast to extrinsic quality factors, most intrinsic factors are more relevant for predicting the quality of experience during consumption [26]. The contributions of factors such as brand and price are likely to decline over time due to fierce domestic market competition, so other factors, such as the sensory quality of beef, will continue to become increasingly important to consumers [5].

#### 2.2.1. Appearance

Consumers can detect differences in quality through the visual appearance of the beef product. Indeed, the appearance of fresh meat is of great importance for consumer purchase [27]. A whole raw steak on display could give the feeling of a good quality premium food. At the time of purchase, meat color and fat color are critical indicators of freshness and quality for consumers [28]. Bright, cherry-red meat color and white fat color are more desirable than dark meat and yellow fat to consumers [29]. In addition, marbling represents the visual appraisal of intramuscular fat (IMF) content and consumer perception of marbling is negative to a large extent due to the sign of excess fat [30], which is not as “trimmable” as preferred by most consumers. Nevertheless, quality preferences vary from one individual to another, Killinger et al. (2004) found that consumers who preferred low marbling appeared to want lean products, whereas those who preferred high marbling favored products of superior eating quality [31]. Overall, when consumers select beef products, they rule out the influence of extrinsic quality traits, with the appearance of the product playing a predominant role at this point. For instance, as it is well known in the beef industry, Pale Soft Exudative (PSE) and Dark Firm Dry (DFD) meat products are unacceptable to consumers in shops due to their appearance defects, which are obviously related to low quality [32]. However, appearance cannot guarantee consistent eating quality at all times. It has been observed that around 15% of the retail beef in some cities in the United States does not meet the expectations of the bright cherry-red lean designation [33]. Additionally, another important factor is the morphological integrity or intactness of the primal cuts; for some consumers, when these muscle cuts are taken directly from the carcass without any processing, this implies naturalness and safety. On the other hand, meat that has been processed, even for the purpose of tenderization, may induce negative consumer attitudes [34].

#### 2.2.2. Technological and Convenience Quality Attributes

Technological and convenience quality attributes are also factors that consumers take into consideration when selecting beef products at the time of purchase. Technological quality is associated with the storage (e.g., shelf life) and processing (e.g., cooking yield) of food, which are influenced by the chemical and microbiological properties (e.g., water/fat holding capacity, antioxidant capacity, bacterial growth) and storage requirements (shelf life, temperature, light, package) of the meat [7]. These quality attributes are also related to the practicality and usability of a product, which is known as convenience quality. A product design based on consumer benefits (e.g., time and effort saving), such as ready-to-cook foods or foods that can be kept at a normal temperature for a long time, would play a positive role in shaping consumer quality perception. Despite these trends, the proper evaluation and indication of technological and convenience quality attributes need to be better explored and formalized [35].

#### 2.2.3. Eating Quality

Beef muscle contains approximately 75% water, and the ability to hold water and bind it in the meat during processing is strongly associated with beef texture and palatability [36]. Due to the limited ability of objective and accurate measurements to capture the variance of meat eating quality from actual consumer eating experience, sensory evaluations of meat by trained panelists or untrained consumers have been developed and are widely used in meat sensory research.

Meat tenderness depends mainly on three primary factors: (1) background toughness related to connective tissue; (2) degree of muscle contraction; (3) integrity/degradation of the myofibrillar structure during aging and tenderization [37]. In early research on beef sensory evaluation, tenderness was assessed by muscle fiber, connective tissue, and IMF characteristics in addition to global tenderness evaluation [38]. The perception of tenderness through direct measurements (consumer and/or sensory panel) includes three aspects: the ease with which the teeth penetrate the meat at first, the ease with which the meat splits into fragments during chewing, and the amount of residue left after chewing. This illustrates the complexity of tenderness in its definition and measurement. Consumer satisfaction with meat tenderness is based on the interaction between the physical/textural characteristics of the meat and the “mouthfeel”—an experience related to the sensations of biting and chewing [39].

Meat juiciness is defined as the perceived amount of juice and the level of lubrication when meat is masticated in the mouth. It is mainly affected by the inherent properties of the meat such as water holding capacity (WHC), fat content, and *pre-rigor* muscle metabolism; the physiological state of the tasters such as taste sensation also has an impact on the perception of meat juiciness. Therefore, as a unique subjective property of meat, a relevant measure of juiciness is achieved by sensory evaluation with consumers and/or panelists. The evaluation of meat juiciness can be performed in two steps: (1) initial juiciness, which is the initial impression of meat fluids released by the first chews of the meat and which is related to the water content of the meat; (2) sustained or overall juiciness, the perception of juiciness during sustained mastication known to be associated with fat content, which is considered to be the result of the stimulating effect of fat on salivary flow with different individual tasters [40]. As early as 1972, meat scientists found that juiciness accounted for part of the variance (less than 19%) in meat texture [41]. In the first Meat Descriptive Sensory Evaluation published by the American Meat Science Association (AMSA), juiciness was used as a key factor in evaluating meat eating quality [42]. With the development of sensory evaluation, juiciness plays a consistent role in meat eating quality. In the MSA system, 10% of the variability in consumer acceptance is explained by juiciness [43]. For American consumers, juiciness accounts for less 10% of the overall palatability of beef [44].

Flavor is a very complex sensation detected by humans, which involves a combination of olfactory and gustatory sensations that detect basic taste and aromas [45]. Physical factors (i.e., breed, sex, and age) and chemical traits (i.e., fatty acid profile) have heavy impacts on the reactions within beef during the cooking process with regard to the production of volatile aroma compounds and the taste of the beef [46]. Flavor has always been considered as an important component of beef eating quality to consumers. Efforts have been made and documented to formally improve beef flavor for more than two hundred years. In the evolution of beef sensory quality research, flavor was included in the sensory description system in 1995 by the American Meat Science Association [47] twenty years after the introduction of tenderness and juiciness. Nevertheless, meat scientists still regarded beef flavor as the second most important attribute for beef eating quality and consumer acceptance, with tenderness being the first most important [48]. In recent decades, in the MSA system, flavor liking has become as important as tenderness [43]. Furthermore, with the improvement of tenderness in recent decades, flavor is considered the most important determinant of variability in beef eating quality [49]. Beef flavor has been expanded to describe specific components such as species-specific flavor (beef broth) or descriptive attributes formed from the Beef Lexicon (fat flavor, bloody, grainy, grassy, cardboard, painty, fishy), and these attributes are related to consumer sensory attributes [46]. In current studies, flavor liking is used in the MSA system with untrained consumers, and typical flavor and abnormal flavor are used in beef evaluation with panelists.

#### 2.2.4. Health Quality

With the improvement of people’s living standards especially in developing countries, and the increase in food safety issues, consumer perception of beef quality is highly influenced by the potential health and nutritional benefits as well as the quality of safety in the daily purchase of meat. Furthermore, with the development of more safety control and traceability systems, consumer perception of meat safety has been improved, in particular with the provision of information on safety supervision [50].

As indicated by Clinquart et al. (2022), microbiological quality is essential for beef safety and health quality. Indeed, foods of animal origin (e.g., beef, chicken, and pork) are major reservoirs of many foodborne pathogens such as Shiga toxin-producing *E. coli*, *Salmonella,* and *Campylobacter* [7]. Illness and even death that are caused by meat-related foodborne pathogens raise great concern for the conventional meat industry [51]. In Europe, the prevalence of *Salmonella* in cattle is about 2% [52]. Bacterial contamination of meat occurs during the muscle to meat conversion, transport and slaughter, processing, storage and cooking, *pre-slaughter* stress is identified as a factor affecting *Salmonella* and pathogenic *E. coli* contamination of animals [53]. A hygienic operating environment on the slaughter floor and chilling are essential elements in controlling all biological hazards. The microbiological issue of beef products can be serious when the meat is raw or undercooked. The meat product, especially processed beef (ground beef), must therefore meet at least the microbiological criteria set out in the relevant regulations [7]. There is no doubt that microbiological quality can affect human health, while synthetic pesticides, antimicrobials, and growth hormones used during animal production to treat infections and prevent diseases and also to optimize growth are another problem that threaten human health [54] and should therefore be rejected for consumption, especially for sensitive individuals.

Meat plays a crucial role in human evolution through the supply of essential macro and micronutrients, including high biological value proteins, fatty acids, iron, zinc, selenium, and vitamins B3, B6, and B12. Many factors such as animal type, farming system, muscle type, processing, and cooking have an impact on the concentration of these macro and micronutrients. Consumers eat meat because it is delicious in taste and necessary for its good nutritional quality [55]. Thus, consumers tend to prefer organic food, which ensures that synthetic fertilizers, pesticides, and hormones are avoided in the production process and that the use of veterinary drugs is minimized [56]. In addition, previous studies have demonstrated that organic beef has higher nutritional value than conventional beef in terms of improved bioactive compound content and a better balanced fatty acid (FA) composition, with a higher level of poly-unsaturated fatty acids (PUFAs) especially n-3 PUFAs [35]. In view of the importance consumers place on nutritional value, they would be willing to pay a premium for organic meat [57], especially for a better composition of beneficial FAs [58]. In addition, concern about chronic nutrition-related complications is in contradiction with the desire to consume meat, which might have a higher fat content for better eating quality.

In general, consumers already perceive meat as a healthy component in their diet. With the evaluation of consumer expectations, an increased interest in credence quality and health quality has been observed, which were identified above as often as being related to the quality of the production process [59]. Consumers consider high animal welfare standards or natural grass feeding to be associated with increased safety, healthiness and eating quality of food [60]. On the other hand, consumers are increasingly concerned about food-related risks and prefer natural foods (i.e., non-invasive technologies or non-chemical processes) to artificially produced foods [61]. Similarly, some consumers are opposed to novel products such as cell-based meat, due to concerns about unnaturalness and high degree of artificial production, with no assurance that cell-based meat will be safe and healthy [62].

## 3. Main Factors Affecting Beef Eating Quality

As one of the most commonly consumed protein sources, beef is an important food in the world [63]. However, a global decline in beef consumption has been observed over the last two decades in Europe [6]. Beef consumption is highly associated with beef eating quality and consumer satisfaction. The complexity of guaranteeing beef eating quality and the inability to select beef with consistent palatability have therefore been regarded as major factors in explaining the decline of beef consumption [64]. This is because beef quality, especially palatability, is characterized by inherent variability and depends on many interacting factors that are complicated to handle, such as ante-mortem factors including sex, age, maturity and breed of animals, carcass fat level, FA composition of cuts, and *post-mortem* factors involving the slaughter process, carcass handling, aging, storage, and cooking [65]. This section mainly focuses on the factors that affect beef eating quality. The ranges of factors affecting each set of core quality attributes are summarized in Table 1.

### 3.1. Antemortem Factors Affecting Beef Eating Quality

#### 3.1.1. Breed

*Bos taurus* and *Bos indicus* are the two main cattle breed groups in the world. They are genetically adapted to survive with high productivity in adverse conditions, including heat, drought, and poor-quality pastures. It is well known that the meat produced by *Bos indicus* cattle tends to be of lower quality [66]. Indeed, some beef cuts from *Bos indicus* cattle can be tough due to the genetical effect on the calpain-calpastatin system, muscle fiber size, and metabolic properties, which result in inhibited protein degradation and ultimately decreased sensory tenderness [67]. This has led some labeling systems to exclude *Bos indicus* meat from their certified brands, thus hindering the presence of *Bos indicus* meat in major markets [68]. In fact, it has been observed that other differences such as IMF deposition and FA profile of meat produced by *Bos taurus* and *Bos indicus* cattle depend mainly on feeding system [69].

Breed-related differences in beef eating quality have long been discussed with respect to grow path and age at physiological maturity, which are mainly reflected in muscle structure, the content and solubility of connective tissue and the amount, and the composition and distribution of adipose tissue, especially IMF in beef [70]. The beef produced by the Wagyu breed, notably characterized by its intense marbling [71], has a more intense flavor and juiciness than that of the Angus breed [72]. Intramuscular adipose tissue matures late and accumulates as the animal grows and matures, with IMF being deposited after intermuscular fat, which is itself deposited after subcutaneous fat [73]. Therefore, at similar levels of maturity, early maturing breeds (e.g., traditional British beef breeds such as Angus and Hereford) have a tendency to deposit more IMF and can be slaughtered at lower weights with a higher fat content, compared to late-maturing beef breeds (e.g., continental European breeds such as French Limousin, Charolais, Blonde d’Aquitaine and Belgian Blue), which have relatively less IMF. With a different level of marbling, the beef eating quality is therefore different for these two types of breeds.

Beef quality is multi-determined and must be analyzed based on many factors to avoid a biased comparison. In general, the eating quality of meat from beef breeds is considered better than dairy breeds. However, untrained consumers reported hardly any differences in eating quality between meat from dairy and beef breeds, except for a few muscles [74]. Although breed has an important effect on beef sensory quality, beyond differences in carcass characteristics, breed might explain only a small part of the variability in beef quality or sometimes may not explain it at all. For instance, Conanec et al. (2021) observed very few differences in beef sensory quality between beef aged under the same conditions and produced by young bulls from 15 European breeds reared under relatively similar conditions [75].

#### 3.1.2. Sex

There are several differences between sex categories related to hormone level and muscle composition and in interaction with genotype [76]. Heifers are identified as more tender than bulls and steers with less intramuscular connective tissue content and smaller muscle fiber diameter [77]. In contrast, bulls grow more rapidly and produce carcasses with less fat and more red-oxidative muscle than steers [78]. Steers are rated less tough and more palatable with more IMF compared to bulls [79]. Moreover, it was found that even after adjusting for different carcass traits, meat from bulls had lower eating quality scores than meat from females and steers [74]. Sex also influences meat color especially in combination with age; female animals tend to deposit more pigment with age than males [80]. However, in general, due to higher physical activity and myoglobin concentrations, the meat from intact males is darker than that of females and castrated males [81]. Nonetheless, in practice, the meat from females usually comes from dairy cows or cull cows slaughtered at a later age, which usually results in a darker color [7].

#### 3.1.3. Animal Age and Maturity

In general, increasing age and maturity is correlated with a decrease in eating quality. With increasing age and maturity of the animal, the collagen content increases and the heat stability of the collagen declines, the shear force and toughness of the cooked beef proportionally increase [82]. Meat color and fat color are generally influenced by animal age, with L* and a* values higher for older animals than younger ones [83]. Meanwhile, older animals tend to contain more fat, and the percentage of IMF increases with a concomitant increase in the percentage of monounsaturated fatty acids (MUFAs) and a decrease in that of PUFAs, and this is associated with better flavor intensity [84], but lower healthiness due to higher proportions of saturated fat. In addition, it was found that the decrease in tenderness appears to be less pronounced with beef from animals over 18 months of age compared to animals under 18 months of age, although this is animal- and muscle-dependent [85]. Moreover, the flavor intensity of beef tends to increase up to the age of 18 months and thereafter reaches a plateau [86]. Kopuzlu et al. (2018) also found for Eastern Anatolian Red bulls that beef tenderness, juiciness, flavor, and overall acceptability increased until the animals reached 19 months of age [83].

#### 3.1.4. Feeding System, Fat Content and Marbling

The feeding system has effects on beef quality since the nutrient composition and energy intake of the diet can affect the animal’s growth rate, degree of maturity, and carcass composition, particularly the amount of IMF and the FA profile [87]. Diet composition and finishing management have different effects on beef quality traits, especially for different animal categories. Specific analyzes are therefore necessary to determine the impact of feeding on beef sensory quality in specific circumstances, such as when animals are inconsistently characterized. In the beef industry, different finishing systems are used, resulting in beef product variations. In general, beef produced in extensive production systems is considered to have a healthier FA composition, and pasture-based feeding strategies are developed for this purpose as consumers prefer grass-fed beef as it is perceived to be healthier and “greener” [88]. However, scientists found that rearing systems (indoor rearing vs. outdoor grazing) had no major impact on Warner Bratzler shear force (WBSF), texture profile, WHC, and most of the sensory attributes of *m. longissimus dorsi lumborum* from Podolian beef [89]. However, in general, due to higher IMF accumulation, grain-finished beef from feedlot systems is perceived as superior to that of grazing systems and/or forage/pasture-finished cattle, which tend to produce leaner beef [90]. In addition, from the perspective of eating quality, some consumers prefer grain-fed/finished beef because pasture/forage-fed/finished beef contains specific pastoral flavors such as “grassy”, “wild” and “barny” and lacks the normal beef flavor [91]. In contrast, forage/pasture-finished beef generally has an increased conjugated linoleic acid (CLA) and PUFA to SFA (saturated fatty acid) ratio [92], which is better for human health, especially in reducing the incidence of many diseases such as heart and cardiovascular diseases. Additionally, pasture quality is an important element in differentiating beef quality. Therefore, meat from pasture-fed cattle may not only be of comparable quality to meat from grain-fed animals [93] but may even be more tender [94].

One of the traits most influenced by feeding practices is IMF, which is well known to affect beef eating quality. It has been reported that grain-finished beef is considered to have a more acceptable flavor than forage-finished beef [95] due to a higher IMF content. The fat content of a beef carcass is composed of adipose tissue deposited in the abdominal cavity (perirenal, mesenteric, and omental), intermuscular, subcutaneous, and intramuscular [73]. Of these, IMF content plays a key role in beef eating quality [96], although the relationships between them may depend on confounding effects such as animal breed, sex, and age, and feeding systems. IMF content refers to the lipid deposit in muscle and is an objective measure of the total triglyceride and phospholipid content present on a microscopic level [9]. The visible portion of the IMF is termed “marbling” and is widely used as an indicator of IMF content and meat quality in beef grading systems in the USA and Australia [70]. While marbling accounts for nearly 75% of the variation in IMF [97], chemical IMF% and marbling level are similar in their prediction of eating quality in *m. longissimus thoracis and lumborum* and other cuts [98].

Fat is not defined as a basic sensory trait but provides meat with specific mouthfeel and lubrication between muscle fibers that could increase the perception of tenderness and juiciness, and in particular provides meat with a flavor profile and aromas [37]. Numerous studies have investigated the relationship between IMF/marbling and beef sensory quality. It has been reported that 10–15% of the variance in tenderness evaluation could be explained by marbling [99], and that 2% to 56% of the variation in flavor could be explained by IMF content [100]. Although no evidence shows that excess fat leads to a progressive increase in flavor and palatability [101], higher IMF content could lead to diminishing returns on beef sensory traits. Undoubtedly, a range of acceptability for IMF and marbling could improve beef eating quality [102]. However, significant but varied associations with sensory quality attributes are often observed as this relationship is highly dependent on confounding factors, including animal breed, age, and sex. Nevertheless, several studies agree that there is a curvilinear relationship between IMF content and beef flavor; flavor intensity increases with IMF content, then reaches a plateau at higher levels of IMF [4,103].

#### 3.1.5. *Pre-Slaughter* Stress

Prior to slaughter, animals are exposed to certain situations that can trigger a stress response that can reduce the eating quality of the meat. Improper handling during transport and at the abattoir can lead to muscle glycogen depletion and inadequate acidification and ultimately high pH, resulting in dark cut beef and reduced sensory tenderness [104], juiciness, and flavor [105]. However, higher pH is not always the only reason for the reduced quality of stressed cattle. This has been confirmed by several studies: *pre-slaughter* stress was found to have a negative impact on consumer-assessed eating quality, even if the ultimate pH of the carcass was compliant (pH ≤ 5.7) [106] and, with a compliant pH, WBSF was higher in stressed cattle [104].

There is no doubt that *pre-slaughter* stress is associated with lower beef eating quality and it has been demonstrated that mixing and transporting animals prior to slaughter was associated with lower eating quality for some cuts and that a two-week rest period in the slaughterhouse prior to slaughter is beneficial in improving consumer perception of beef sensory scores [107]. The beef industry and some quality grading systems, such as MSA, have developed pathways to minimize the adverse effects of physical activity and emotional stress prior to slaughter. For instance, different lairage periods are recommended according to the transport journey to enable animals to rest, rehydrate, and replenish their glycogen stores [108]. In the MSA system, some *pre-slaughter* pathways that may maximize stress could be penalized such that cattle sold in the saleyard prior to slaughter are deducted 5 points from the final MSA meat quality score [43].

### 3.2. Post-Mortem Factors Affecting Beef Eating Quality

#### 3.2.1. *Post-Harvest* Aging and pH/Temperature Decline

*Post-harvest* aging is a value-adding process which involves storing the carcass at cold temperatures for varying periods of time, profoundly affecting the biophysical and biochemical modification conditions of the carcass through regulating *post-mortem* energy metabolism, proteolysis, and apoptosis [109]. These processes lead to a progressive increase in tenderness and flavor with the disintegration of muscle structure and the release and accumulation of peptides and free amino acids. Based on theoretical knowledge, several practical adjustments could be implemented to improve beef palatability with some treatments such as aging, with some breeds showing optimum tenderness at short aging periods and other breeds requiring longer aging to achieve similar consumer acceptance [110]. Several beef grading systems use aging time as a parameter to guarantee/predict beef quality. According to MSA, five days of aging is required as a minimum aging period; for the French Label Rouge, ten days is generally considered [91]. Longer aging times up to a certain level are generally good for better palatability; for *m. longissimus dorsi*, it takes 4.3 and 10 days to reach 50 percent and 80 percent of total aging, respectively. The aging process affects muscles differently: slow-twitch muscles are thought to age more slowly than fast-twitch muscles [111]. The tenderness of *m. psoas major* and *m. semitendinosus* needs 7 and 14 days to improve, whilst *m. longissimus lumborum* can achieve the most tender score at 21 days [112].

Extensive consumer studies have shown that beef eating quality is negatively affected when the carcass enters *rigor mortis*, which refers to contraction of muscle fibers [113]. If the temperature drops too quickly (below 12 °C) and the pH is high (above 6) during rigor, this combination known as “cold shortening” could cause muscle contractions that could increase toughness by up to four times. In contrast, the combination of a high temperature of carcass (above 35 °C) and rapid pH decline (below 6), called “heat shortening” could cause muscle protein denaturation and muscle shortening, which could lead to increased meat toughness and dryness [114]. Therefore, the control of pH and temperature decline within the optimum pH/temperature window (pH of 6 between 15–35 °C) would be an effective way to limit the extent of muscle shortening and optimize eating quality. Meanwhile, after muscle toughening, tenderization also takes place during the *post-mortem* period [115] based on certain biochemical reactions such as proteolysis. Among these, calpains play a primary role in meat tenderization during *post-mortem* aging and could be optimally activated under regulation of the physiological pH of skeletal muscle [116]. Calpain activity is optimized with an intermediate pH decline to 6.0 at 1.5 h *post-mortem* [117]. In addition, being compatible with pH, temperature plays a key role during *post-mortem* aging, with a too-low temperature having negative impacts on tenderization by slowing down enzyme activity. Meat eating quality was found to be greatly improved when carcasses reach 21 °C at pH 6 [109]. Aging of 86% can be achieved when carcasses are held at 30 °C for 24 h [118]. Nevertheless, for microbiological growth and food safety reasons, high temperature aging is not practically useful for the industry. Therefore, an optimal pH/temperature intervention can be conducted *post-mortem* to optimize the tenderization.

Aging methods can generally be classified as wet and dry or a combined method in a stepwise dry/wet aging process. Dry aging is less applied than wet aging due to higher cost, more stringent operational requirements, lower sealing yields, and longer aging time. However, in contrast to the “wet-aged flavor” which is sour, metallic, and bloody, dry aging is increasingly appealing to consumers because of the perceived “dry-aged flavor” as nutty, roasted, or butter, which is due to the concentration of typical aroma compounds that dry aging provides. Dry aging is also found to improve the eating quality of beef with a lower marbling level [119].

#### 3.2.2. Electrical Stimulation (ES)

Two mechanisms could explain the effect of ES on tenderization. The primary effect is to reduce cold shortening by accelerating glycolysis and rapid pH drop to avoid the temperature drop at which cold toughness occurs [120]. The secondary effect is to accelerate proteolysis by stimulating the release of Ca ions at a higher temperature [121] and to increase disruption of muscle structure [91]. Based on these two effective tenderization mechanisms, ES has therefore been applied in the worldwide meat industry for decades to achieve optimal tenderization, especially in combination with pH/temperature controls. It has been reported that when carcasses were electrically stimulated and held at 35 °C for 3 h, a fast drop in pH to 6 and significant increases in μ-calpain activity and ultimately in tenderness were observed [122]. In addition to the beneficial effect of ES on tenderness, some improvements are observed regarding juiciness and flavor and overall satisfaction [123], as the perception on those sensory traits in electrically stimulated meat is more impacted by the fat content [124].

The voltage of ES has long been investigated, with the use of high-voltage ES (3600 V) being first investigated [125], followed by low-voltage ES (32 V) [126], which was more used in the industry due to safety concerns. In fact, high and low voltage ES with different durations can achieve the same tenderization effect [127]. Recent research has focused on combining chilling methods (Tenderstretch, super chilled storage, and long aging time) with new technologies such as the so-called new generation medium voltage ES. The tenderness of meat subjected to medium voltage ES has been improved due to various reasons such as physical disruption of the muscle structure [128] and myofibrillar degradation [129].

#### 3.2.3. Carcass Suspension

Several hanging methods have been used to improve meat tenderness during *post-mortem* aging. Achilles tendon is the most traditional and widely used carcass suspension method, although it cannot prevent the majority of muscle shortening, but, with the appropriate aging process, Achilles tendon still can achieve the tenderization potential of beef cuts [91]. In comparison with Achilles tendon, Tenderstretch increases tension and results in more tender meat, but this varies between muscles, with improved eating quality in most hindquarter muscles [130]. In general, different muscles could respond differently to *post-mortem* aging and, therefore, muscle-specific aging strategies could improve tenderness and overall eating quality [112]. In fact, Tenderstretch could effectively shorten aging time and improve beef tenderness by up to 40% [131], and indeed performs better on improving beef sensory quality (flavor, juiciness, and overall liking) than that of Achilles tendon [132].

## 4. Main Methods for Measuring and Predicting Beef Quality

In order to ensure in advance a good quality of beef at consumer level, a relevant method is to predict beef quality in scenarios where the beef carcass and muscle cuts can be evaluated by combining different quality traits (for instance at farm level, at abattoir level, or by some specific approaches such as consumer sensory testing). Perceived quality, particularly at the time of tasting, depends on a combination of parameters that have been largely evaluated by consumers or more generally by human panels in recent decades of research. In fact, many traits are initially and still largely measured by objective methods [120]. In the beef sector, there are mainly three categories for beef quality measurement/prediction: (1) instrumental methods (intrusive mechanical measurement and non-destructive instrumental measurement), (2) the omics approach, and (3) the carcass/cut grading schemes [6].

### 4.1. Mechanical Measurement of Beef Quality

#### 4.1.1. Physical Texture Measurement

Evaluation of beef quality is complicated, especially with respect to sensory quality, which in reality can only be measured by consumers or sensory panels [133]. However, since consumer evaluation is time-consuming and costly, it cannot be widely used for all quality measurements. A widely-used method of evaluating meat quality is to measure the physical texture of meat products. The physical texture of beef is mainly related to mechanical attributes, which are generally characterized by hardness, cohesiveness, viscosity, springiness, and adhesiveness [134]. Mechanical measurements of the strength required to break down the meat are mainly categorized as shearing, biting, compressing a standardized piece of meat. The most commonly used measurement for meat toughness/tenderness is the WBSF. The Slice shear force (SSF) is a faster alternative to WBSF but is less used [135]. For overall physical texture, there is the texture profile analysis (TPA), and some devices are used such as the MIRINZ tenderometer with a biting action for measuring overall tenderness of meat [133]. The WBSF was found to be more effective in classifying beef as tender (68% accuracy) than the SSF (47%), compared to consumer perceived sensory tenderness (80%) [136]. Many studies have tried to relate the meat physical texture measurement to consumer-rated tenderness/mouth-feel-taste, with physical measurements being able to explain a variable variation in tenderness assessed by human panels but no more than 60% [133]. Platter et al. (2003) found that WBSF can only explain 23% of the total variance of consumer-scored tenderness [137]. Various correlations between WBSF and consumer evaluated tenderness have been observed, ranging from low (e.g., r = −0.19, −0.26) [138] to high values (e.g., r = −0.72, −0.82) [139]. Different factors such as aging process, cooking temperature [140], and muscle cuts [141] might contribute to these inconsistencies. Except for the above, the lack of strong correlations between physical shear force and consumer-perceived tenderness indicates that they seem to be two non-equivalent issues, the latter being not only related to mechanical force but also associated with sensations generated by moisture and fat within the meat.

#### 4.1.2. Juiciness Measurement

According to a National Beef Tenderness Survey conducted in the United States at the food service and retail level, over 94% of rib and loin beef were rated tender or very tender. Such a large proportion of tender beef has magnified the importance of juiciness and flavor to the consumer eating experience [142]. This is the reason why the importance of beef sensory traits has renewed attention from meat scientists in recent years. For many years, tenderness was considered as the dominant factor in determining eating quality and with the clarification of a higher contribution of flavor liking to overall consumer satisfaction, the importance of juiciness should not be neglected [49].

The measurement of juiciness has previously focused on total water content, WHC, and water fractions of meat, although the consistency between sensory juiciness and these parameters varies [143]. One of the reasons could be that the meat evaluated by consumer or panels has been cooked, which means that with physical/chemical alterations and intra- and extra-myofibrillar water movements, the perception of juiciness may be altered. Cooking loss, drip loss, and compression-based methods have been usually used to quantify expressible moisture in meat. Cooking loss has been reported to be able to explain 60–80% of the juiciness variance [144], but it has also been reported that cooking loss cannot explain the juiciness of cooked meat due to heat-induced changes [143,145]. Compression-based methods have evolved from filter paper press methods from the Carver hydraulic press apparatus, the Instron-based press method to the pressed juice percentage (PJP) method with various capabilities to predict juiciness scored by a sensory panel [146]. PJP was observed to be strongly correlated with sensory juiciness scored by trained and untrained consumers (r = 0.69, 0.45), respectively. IMF content can also be a good indicator of juiciness. Thompson (2004) found that consumers were satisfied and dissatisfied with beef juiciness when IMF was above 20% or below 2%, respectively [103]. However, it is difficult to define a threshold of juiciness for consumer perception based on IMF content due to the different distribution of IMF [147].

#### 4.1.3. Flavor Measurement

Flavor is perceived by consumers through two pathways, namely odor detected by the nose and taste perceived by the mouth and tongue. There are receptors on the olfactory bulb in the nose and mouth that detect volatile compounds; when they come into contact with the olfactory bulb and are recognized by these receptors, flavor is thus perceived. In addition to these volatile compounds, there are volatile aromatic compounds generated in the mouth during chewing or swallowing of meat. However, the amount or types of receptors and the amount or concentration of volatile compounds needed for perception vary between individuals [86]. The perception of flavor is therefore complicated to define due to the individual diversity of the taster. This is the reason why meat flavor is further described and assessed by highly trained descriptive attributes with panelists, which are the most accurate methods for measuring meat flavor.

Mechanical measurement of flavor on the basis of consumer perception is challenging due to the complexity of the meat matrix and consumer perception. In recent decades, significant progress has been made in identifying and quantifying meat flavor compounds [148]. Thousands of volatile compounds have been identified as constituting the aromas of meat odor/flavor using mechanical and/or chemical measurements such as olfactometry, flame ionization detection (FID), and thiobarbituric acid reactive substances (TBARS). TBARS have been shown to have a predictive ability for the consumer’s flavor liking threshold, but this is highly dependent on the method used for TBARS determination. FA profile can contribute to consumer flavor liking, as CLA, SFAs, and MUFAs have been associated with flavor liking, although some effects are muscle-dependent [149]. Additionally, the electronic nose (e-nose) and electronic tongue (e-tongue) are also useful tools for evaluating meat flavor attributes [150].

### 4.2. Non-Destructive Instrumental Methods for Beef Quality Prediction

There has been a demand to predict beef quality by non-destructive instrumental methods, which are considered as having many clear-cut advantages, such as ease of use, non-destructiveness, speed, cost-effectiveness, reproducibility, and a high potential accuracy [12]. Ongoing work with various emerging technologies has been conducted with the aim of predicting beef quality directly or indirectly, i.e., predicting consumer sensory attributes directly related to quality, such as tenderness or flavor, or predicting indirect quality-related parameters that have been shown to have an impact on meat quality such as meat color, pH, IMF content, or marbling [6].

The use of Near-InfraRed Spectroscopy (NIRS) to predict the chemical composition, technological parameters, and sensory feature, of meat quality attributes, such as WBSF values and trained panel or untrained consumer sensory scores, is a topic with important applications in meat plants, as both WBSF and sensory measurements are time-consuming and destructive; however, due to the complexity of predicting these attributes, the determinant coefficients proposed in the literature are variable. NIRS can correctly detect 80–95% dark cut beef depending on the instrument used [6]. Several studies have suggested that the sensory quality of meat can be accurately predicted by NIRS but with relatively low accuracy (R^2^ = 0.10–0.58) [151,152], although Ripoll et al. reported that beef tenderness could be predicted by NIRS with high accuracy (R^2^ = 0.98) [153]. Computer vision techniques have been utilized to visually assess meat quality in the processing line as they are non-invasive and consistent to assessing color, IMF and, most importantly eating quality [154]. It has been reported that computer vision has the ability to assess marbling and predict quality attributes with R^2^ values for tenderness (0.72), WBSF (0.83), juiciness (0.60), flavor (0.78), and overall consumer acceptability (0.82), respectively.

Hyperspectral imaging is a more promising technique for the objective assessment of meat quality attributes such as color, tenderness, and texture. Through an integrated system of spectroscopy and imaging techniques, images of the entire sample surface can be recorded, thus reducing the negative effect of non-uniform distribution of meat constituents. Several studies have demonstrated that hyperspectral imaging technique can predict meat tenderness and WBSF quite well, with R^2^ values of around 0.9. With appropriate statistical methods such as discriminant analysis, the classification of tenderness between tender and tough meat can reach an accuracy of 75% to 96% [155,156].

### 4.3. Omics Approaches

#### 4.3.1. Genomics

The criteria for defining consumer beef eating quality are based on several traits (e.g., tenderness, juiciness, and flavor, etc.), which are quantitative traits determined by sets of components regulated by the joint action of numerous genes and environmental regulations (growth, rearing and processing factors) [157]. Each individual component contributing to the palatability phenotype is consequently difficult to control and costly to measure. All beef eating quality traits are difficult to improve based merely upon phenotypic selection, but there may be effective candidate genes for genomic selection if genetic markers that account for a significant variance for those quality traits are identified [158].

Within the meat sector, numerous genes have been identified as being involved in valuable estimates of genetic parameters. They provide key insights into the regions that underpin variation in physical meat characteristics, including muscle fibers, connective tissue, IMF, meat color, fat color, shear force, and sensory meat quality traits such as tenderness, juiciness, flavor, chewiness, etc. [159]. So far, some sensory-related traits including tenderness and color have been confirmed with notable representations of related biomarkers on chromosomes [119]. Despite its relevant potential to predict meat quality variation, some limitations have still been noted, the most common being that, thanks to numerous association studies, predictive information can be obtained but not deep scientific knowledge of the underlying mechanisms, at least in the earliest stages of omics development. Moreover, predictive reliability appears to be less consistent, in particular with human-evaluated meat eating quality. For instance, recent heritability estimates for tenderness, juiciness and flavor scores range from 0.1 to 0.2 [160]. This indicates that the proportion of variability in beef eating quality explained by genetic factors is moderate to weak.

#### 4.3.2. Proteomics

An emerging body of literature has examined the proteomic pathways involved in meat eating quality variations [161]. All these works also contribute to the elucidation of the biological mechanisms involved in muscle to meat conversion and in meat qualities [162]. Despite the many factors regulating beef eating quality, and therefore the large number of biomarkers involved in the regulation of quality by these factors, with more and more results from proteomic studies, robust candidate biomarkers can still be identified due to their consistent associations with meat qualities. Gagaoua et al. (2019) found some biomarkers that related to muscle structure (MyHC-I, MyHC-IIa, MyHC-IIx), oxidative stress (DJ-1, PRDX6), and proteolysis (CAPN1) that were consistently associated with tenderization of *longissimus thoracis* muscle. Despite various results depending on animal breeds (Aberdeen Angus, Limousin, and Blond d’Aquitaine), end-point cooking temperature of beef (55 or 74 °C), and consumer origin (France and UK), some of these biomarkers performed as robust predictors for tenderness [163]. Protein network research has revealed the functional annotation of 124 proteins in the *longissimus dorsi* muscle, which are crucial in the production of high-quality beef [119]. More and more integrated proteomics studies have been carried out to create a repertoire of biomarkers, especially for beef quality defects (i.e., dark, firm, and dry beef). The ultimate goal of these biomarkers is to guarantee the eating quality for consumers by proposing a list of validated biomarkers for the development of routine bioanalytical tools to be used by breeders and producers to improve the potential merits of breeds and to detect potential quality during the *pre-* and *post-mortem* periods [119].

#### 4.3.3. Metabolomics

Skeletal muscle is characterized by a set of functionally cooperative genes designed to address the spatiotemporal requirements of each muscle. Gene expression is then regulated, including protein modification, during muscle development, growth, and maturation. In the later stages, muscle metabolites determine the muscle characteristics, which are the major phenotypic components of meat eating quality. During the development and physiological specialization of muscle, many well-known factors all impact on the genome, transcriptome, and proteome profiles of muscle, making it very difficult to understand the precise mechanisms behind meat quality variations through these molecular markers [164]. Nonetheless, changes in muscle metabolome profiles (small hydrophilic molecules/metabolites such as polyphenols, organic acids (carnitine, creatine, and carnosine), amino acids, vitamins and minerals, etc.) can be quantified by metabolomics as potential indicators reflecting the metabolic process and screened to predict sensory quality [165]. For example, Ma et al. (2017) reported that an increase in the amount of free amino acids was associated with the degree of proteolysis, which suggests more tender meat, but also with more precursors of aromatic compounds that play a role in the sensory aspects of cooked meat [166]. Furthermore, Antonelo et al. (2020) found a positive correlation between carnitine and consumer acceptance of beef steaks, while strong negative correlations were observed between carnitine and creatine and consumer sensory scores for tenderness, juiciness, and overall liking [167].

### 4.4. Grading Schemes for Beef Eating Quality

With the advancement of international trade of beef carcasses, carcass classification standards and beef quality grading schemes are required to provide a description of carcasses and muscle cuts with the definition of quality to purchasers and destination markets [168]. Based on this objective, two categories of grading schemes, based on carcass and muscle cut, have been used to classify carcasses and predict beef quality.

#### 4.4.1. Carcass-Based Grading Schemes of Beef Quality

A small number of countries have carcass grading schemes to directly predict beef eating quality. Most of them focus more on a generic scenario of beef quality in relation to carcass characteristics. The current carcass-based grading systems in these regions (mainly Europe, USA, and Japan) primarily encompass two categories of carcass classification, namely yield and quality grading.

Yield is determined by various criteria depending on the system but basically can be defined as lean or saleable meat yield and can be determined by carcass weight and composition. In the USDA (United States Department of Agriculture) system, the yield grading is an indication of yield of boneless, trimmed retail cuts. The JMGA (Japanese Meat Grading Association) yield grade refers to the proportion of meat produced by the animal that can be eaten and is determined by eye muscle area, rib thickness, cold left side carcass weight, and subcutaneous fat thickness through a regression calculation [169]. In contrast with the USDA and JMGA systems, which have a parallel quality evaluation criterion related to beef palatability, the European classification system places emphasis only on the description of production yield rather than beef eating quality. The EUROP grid is established to classify carcasses according to the assessment of carcass weight, muscle shape, and fat level, described by conformation score and fat score, respectively [170]. Since the EUROP grid is widely applied and regarded as traditionally important for the European beef industry, carcasses are assigned and traded to differentially priced sales markets according to the European classification scores [171]. However, meat experts have gradually become aware of the weakness of the EUROP grid nowadays within Europe, as European classification scores have little relation to eating quality at consumer level and cannot reflect carcass composition [172] and consumer satisfaction [173].

Carcass maturity and IMF level (marbling) are two major attributes that are used for quality segments. For example, according to the combinations of maturity and marbling level, carcasses can be graded into one of eight categories as in the USDA system. Maturity indicates the physiological age of the animal (ossification, dentition) rather than the chronological age. The amount and distribution of marbling on the *m. longissimus dorsi* are critical assessments in most carcass grading systems due to the strong association between marbling score and beef palatability. In the USDA, graders evaluate marbling between the 12th and 13th ribs, but in the JMGA, carcass grading is performed at the rib site, between the 5th and 6th ribs [174]. Recently, carcass grading with marbling assessment was conducted in a French private meat plant. This study found no significant difference in marbling score between the 5th and 10th ribs, such that marbling score could be measured at the quarter carcass level [175]. This could provide a theoretical basis for the introduction of marbling score in the European carcass grading system.

Since eating quality varies depending on the cut, carcass-based grading systems lack some degree of accuracy and consistency. Studies of consumer preference for beef eating quality based on the USDA quality grade have shown a far less relevant relationship with consumer preference between USDA grades [176]. Recent studies have also indicated that American consumers were unable to detect differences in eating quality between different USDA grades for tenderloin steaks [177]. In addition, it has been observed that the USDA maturity grade has no impact on eating quality for grain-finished cattle up to the age of 30 months and that only marbling grade is important for eating quality [178]. In fact, in the early days of the MSA system, it was found that carcass grading using only carcass parameters cannot predict the eating quality of a carcass from a variety of production systems. Besides, different muscle cuts have different eating quality for different carcasses, and quality also varies as a result of multiple factors [91].

#### 4.4.2. Cut-Based Grading Scheme—The MSA Grading System

Unlike the aforementioned carcass grading schemes, the MSA grading system is a beef eating quality grading system, aimed at delivering an eating quality guarantee to consumers. There are two ways in which the MSA system differs from other grading schemes: (1) the grading of beef quality is based on each of the MSA muscle cuts rather than the whole carcass; (2) the definition of eating quality depends on the responses of untrained consumers [168], and actual consumer performance has been shown to be consistent with a high degree of accuracy when tasting samples with a wide range of quality variance [179].

In the MSA prediction model, different Critical Control Points (CCPs) have been used from the breeding, production, *pre-slaughter*, processing, and value-adding aspects of the supply chain that have an impact on eating quality. In addition, consumer preference is evaluated through large-scale consumer testing [168]. The parameters used in the prediction model are based on inputs including: animal type and production (*Bos indicus* content, hormone growth promotants, milk fed veal classification and sex, sale yard, and selling method), carcass characteristics (carcass weight, ossification score, hump height, USDA marbling score, rib fat depth, and ultimate pH), *post-slaughter* factors (hanging method and aging time), the prediction of beef eating quality being provided for different muscle cuts, and various cooking methods.

Another crucial part of the MSA system is the extensive use of sensory testing of beef by untrained consumers to develop a combined eating quality score (MQ4, 0–100) based on tenderness, flavor liking, juiciness, and overall liking. To link the carcass characteristics with consumer valued palatability, carcass production and grading inputs (that are statistically related to this combined eating quality score (MQ4)) are combined to form eating quality prediction algorithms for specific muscle cuts (39 cuts in total) in combination with a defined aging period and one of eight different cooking methods [43]. Meanwhile, beef samples are graded by consumers as Unsatisfactory (2 star), Good Everyday (3 star), Better Than Everyday (4 star), and Premium quality (5 star). These categories should correspond to the MQ4 score, and this connection enables the muscle cuts to be allocated to these four quality grades [43]. Consequently, beef can be classified into grades that correspond to consumer expectations. The consumer’s willingness to pay for these grades has been estimated from consumers’ answers: if a 3-star beef is set at a unit monetary value of 1, then 2-star, 3-star, 4-star, and 5-star quality graded products can be subsequently valued at 0.5, 1, 1.5 and 2 respectively [2].

The MQ4 score can be used to reflect the overall consumer eating experience of a muscle cut [43]. The eating quality value of a whole carcass, termed MSA Index, can also be predicted in MSA grading scheme. The MSA index is the sum of the weighted MQ4 scores of all MSA cuts (39 muscle cuts), where the weighting of each cut was calculated as the percentage of the total weight of the MSA cuts in the carcass. The MSA Index is used to value the potential eating quality of beef carcasses and enables producers to monitor the impact of genetic and breeding practices and management on the eating quality of each carcass [180].

#### 4.4.3. The Future Grading Scheme for Beef Palatability in Europe and Other Countries

There are various grading schemes to evaluate beef quality with different standards. In addition, different rearing and feeding systems, environmental conditions, animal type, breed, and processing practices add to the variability of quality evaluation between countries. However, for scientific research on meat quality evaluation for further industrial applications, there is a need to develop and/or share a set of generic principles and/or establish an international database containing a significant number of assessments on beef quality traits from different countries [181]. The MSA protocol has always been considered as a good standard with critical steps including rigorous beef carcass assessment and untrained consumer evaluation of beef palatability. Over the past two decades, independent and/or collaborative studies have concluded that this consumer-focused and cooking- and cut-based quality grading scheme is applicable for many countries such as Ireland, the United States, South Korea, Northern Ireland, Japan, France, Poland, South Africa, and China. It would therefore be very useful to have a platform for comprehensive data pooling and analysis to maximize research efficiency for the benefit of the global beef industry [181].

To this end, a collaborative, non-profit, and independent foundation, the International Meat Research 3G Foundation (3G Foundation), has been established to improve consumer satisfaction of beef quality by promoting worldwide collaborative meat research throughout the bovine supply chain. The platform is designed to coordinate and support global scientific research on beef quality evaluation and prediction by collecting a large amount of data based on a standard methodology (MSA) for further data sharing and modeling and ultimate integrative investigations on beef quality prediction.

#### 4.4.4. Advanced Technology in Consumer Perception of Beef Quality

Human sensory evaluations are applied as useful tools for generating data for the description, discrimination and prediction of meat eating quality. However, time and cost constraints, as well as the lack of flexibility required for successful commercialization, make human sensory testing unsuitable for today’s rapidly changing industrial environment [119]. Moreover, the data generated by human evaluation shows great variability between individuals and the information required for quality perception is becoming increasingly complex as consumer purchasing decisions become more sensitive to both intrinsic and extrinsic factors, including nutritional quality and safety, animal welfare, and environmental and agroecological sustainability. Hence, human sensory evaluations are to some extent heterogeneous methods for generating meat eating quality data based on different emotions, attitudes and responses that are influenced by different intrinsic and extrinsic cues. To better reflect real-world consumer assessments, with the expansion of beef industry into emerging markets, there is a trend to develop and adopt novel and rapid sensory techniques (i.e., Check All That Apply, Napping, Flash Profile, Temporal Dominance of Sensations) to produce data from conventional methods (Quantitative Descriptive Analysis) [182] in order to better understand complex consumer perceptions. Virtual reality is also being used as a tool to improve the analysis of consumer perception on food quality with more realistic parameters through the measurement of consumer psychological and physiological responses [182]. Overall, in order to reduce the variability due to human involvement in meat quality definition and to increase the efficiency of meat quality prediction, novel advanced techniques and methods have been explored and implemented in a more holistic way, taking into account various aspects of consumer quality perception. More information on consumer perception and prediction of meat quality would help to establish a greater degree of accuracy in this area.

## 5. Conclusions

Beef is an essential part of the human diet. Providing consistently good quality of meat is of importance to consumers, which generally implies the production of safe, healthy, and tasty beef. However, meat, especially beef, has gradually become an ideological battleground over the past decades. Producing meat in an environmentally sustainable and animal welfare-friendly manner is therefore critical for the continued success of the conventional meat industry. Beef quality is a complex concept and tends to become more broadly based on different dimensions. Among these, extrinsic quality traits, which include not only factors external to the product but also factors that add value to the product such as animal welfare and environmental sustainability, are becoming increasingly important to consumers.

Intrinsic beef quality traits such as eating quality are of paramount importance to consumer perception of a beef product. However, beef eating quality has a multifactorial determinism with various *pre-* and *post-mortem* factors that have a direct or indirect impact on the ultimate palatability of the beef product. These determining factors include endogenous factors of the animal, rearing and handling conditions, and *pre-slaughter* and *post-mortem* management. Due to the complexity of the determinism of beef eating quality, several approaches (mechanical measurements, non-destructive instrumental methods, and omics approaches) have been developed to assess and predict the eating quality of beef. Despite this, the prediction of quality remains poor at industry level, since some grading systems are simplistic and based on carcasses rather than muscle cuts and most importantly, beef quality should be ultimately appraised by real people. This is why sensory evaluations by untrained consumers and panelists have been widely applied in beef eating quality studies in recent decades. However, so far, no perfect system has been applied worldwide, despite several grading systems, which are well known but mainly implemented locally rather than globally. Therefore, establishing a robust prediction by using more global and accurate approaches, including modelling approaches based on all the above quality determining parameters, would be an efficient solution to guarantee beef eating quality and avoid consumer dissatisfaction. This would be a useful and powerful solution provided that international collaborations could be widely developed.

## Figures and Tables

**Table 1:** Factors affecting the major quality attributes of beef at various stages of the farm-to-fork continuum.

Stage	Factors	Beef Quality Attributes							
		Intrinsic Quality						Extrinsic Quality	
		Appearance	Sensory	Nutritional	Safety	Technological	Convenience	Image	Commercial
Animal	Breed/Genetics								
	Gender								
	Age								
Farming	Feeding—grass/grain								
	Grazing-indoor/outdoor								
*Pre-slaughter*	Transport/load/mix/rest-stress								
Slaughter	Slaughter practices								
	Hygiene								
	Animal welfare								
Carcass handling	Electrical stimulation								
	Aging time/temperature								
	Hanging								
Carcass characteristics	Carcass weight								
	Conformation								
	Marbling								
	Maturity/ossification								
	Rib fat thickness/fat cover								
	Hump height								
	Temperature/pH								
	Meat color/fat color								
Meat	Muscle cut								
	Nutrients: proteins/FA/minerals and vitamins								
Meat products	Packaging/portioning/shelf life								
	Brand/origin/label/grade and traceability								
	Ethical and environmental sustainability								
Processing	Storage								
	Cooking/smoking/fermentation								

In black: major/direct factor of variation; in grey: weaker/indirect factor of variation; in white: not a factor of variation. This analysis is based on the studies of Clinquart et al. (2022), Prache et al. (2021), [7,35] and expertise of authors.

## Data Availability

Not applicable.
